# Superconducting Quantum Magnetometer Based on Flux Focusing Effect for High-Sensitivity Applications

**DOI:** 10.3390/s24123998

**Published:** 2024-06-20

**Authors:** Antonio Vettoliere, Carmine Granata

**Affiliations:** Consiglio Nazionale delle Ricerche, Institute of Applied Sciences and Intelligent Systems, 80078 Pozzuoli, Italy; antonio.vettoliere@cnr.it

**Keywords:** dc SQUID, magnetometer, magnetic field noise, Josephson junctions, integrated device

## Abstract

A superconducting quantum magnetometer for high-sensitivity applications has been developed by exploiting the flux focusing of the superconducting loop. Unlike conventional dc SQUID magnetometers that use a superconducting flux transformer or a multiloop design, in this case, a very simple design has been employed. It consists of a bare dc SQUID with a large washer-shaped superconducting ring in order to guarantee a magnetic field sensitivity B_Φ_ less than one nT/Φ_0_. The degradation of the characteristics of the device due to an inevitable high value of the inductance parameter β_L_ was successfully compensated by damping the inductance of the dc SQUID. The size of the magnetometer, coinciding with that of the washer, is 5 × 5 mm^2^ and the spectral density of the magnetic field noise is 8 fT/√Hz with a low frequency noise knee of two Hz. The excellent performance of this simple magnetometer makes it usable for all high-sensitivity applications including magnetoencephalography.

## 1. Introduction

New quantum technologies, developed within the so-called “second quantum revolution”, represent a strategic sector that has attracted great scientific interest. Particularly interesting are quantum sensors, thanks to which it is possible to carry out both basic studies and highly sensitive applications [1]. Typical measurements that have an impact on both scientific theories and technological applications are those of time and rotation, but also of magnetic fields, accelerations, and speed.

In the field of high-sensitivity magnetometry, the investigation of the magnetic properties of matter at nanoscale level [2,3,4] and the functional imaging of the brain through the measurement of the very weak magnetic fields generated by neuronal currents [5,6,7] are particularly interesting. For these studies, the most used quantum magnetic sensors are Superconducting Quantum Interference Devices (SQUIDs) [8,9,10,11], optically pumped atom magnetometers [12,13], and diamond magnetometers based on nitrogen-vacancy centers. [14,15]. SQUIDs are the oldest and most consolidated quantum magnetic sensors, but at the same time, they are the most sensitive sensors capable of carrying out measurements with an energy resolution at the quantum limit (few Planck’s constants per unit of bandwidth) [16,17]. Thanks to these peculiarities, SQUID devices are employed in many fields such as magnetoencephalography, nanomagnetism, geophysics, magneto-telluric investigations, the non-destructive analysis of materials, magnetic microscopy, metrology, the readout of highly sensitive detectors (transition-edge sensors and cold-electron bolometers), and basic physics investigations [2,9,10,11,18,19,20].

In this article, we will deal with a dc SQUID which, in its simplest form, consists of a superconducting ring interrupted by two Josephson junctions. If the device is biased with a constant current just above the Josephson critical current, the voltage output of a dc SQUID is a periodic function of the magnetic flux linked to the superconducting ring whose period is the flux quantum Φ_0_ = h/2e = 2.07 × 10^−15^ Wb = B S, where B is the magnetic field normal to the loop surface and S is the loop area. Since Φ_0_ is a constant, by increasing the loop area, the magnetic field sensitivity increases. However, the design of a dc SQUID device depends on the type of application we intend to carry out. For example, if high magnetic field sensitivity is required, such as in the case of magnetoencephalography, a dc SQUID magnetometer design will be based on a superconducting flux transformer [21,22,23] or a multiloop configuration [24,25], since it is not possible to merely increase the geometric area of the superconducting loop without performance degradation in terms of both the magnetic flux–voltage transfer factor V_Φ_ and spectral density of magnetic flux noise S_Φ_^½^. In particular, the V_Φ_ and the S_Φ_^½^ decreases and increases with the inductance of the dc SQUID loop, respectively [26,27]. It is therefore necessary to use more complicated designs which avoid an increase in the dc SQUID inductance and at the same time provide an effective flux capture area big enough to achieve magnetic field sensitivities of the order of a few fT per bandwidth unit. In the case of a superconducting flux transformer, a square superconducting coil (pick-up coil) is typically connected in a series with a superconducting multiturn coil (input coil), which is magnetically coupled to a dc SQUID coil in a washer shape. When a magnetic flux Φ_p_ is applied to the pick-up coil, due to the Meissner effect, a screening current flows into the pick-up coil to nullify the total magnetic flux. Such screening current also flows in the input coil, inducing a magnetic flux Φ_S_ into the dc SQUID loop. From simple calculations, it is possible to verify that the magnetic field necessary to link a flux quantum in the dc SQUID loop is lower than that used to perform the same function directly in a dc SQUID loop. In this way, it is possible to increase the effective area of the dc SQUID up to about 3 mm^2^, which is 50 to 60 times the dc SQUID loop geometrical area. In the multiloop configuration, *n* pickup loops are connected in parallel (reminiscent of a wheel with spokes), so that the total dc SQUID inductance is L ≅ L_p_/n^2^ and the total effective area is given by A_eff_ ≅ A_p_/n, where L_p_ and A_p_ are the inductance and the geometrical area of the single pickup loop, respectively. Such a scheme allows us to obtain an effective area of more than 3 mm^2^, keeping the dc SQUID inductance at a reasonable value.

A simpler alternative design could be very useful from both a fabrication and application point of view, especially for those applications requiring many dc SQUID sensors.

In this paper, we report results concerning the design, the fabrication, and the characterization of a high-sensitive dc SQUID magnetometer based on a very simple design without using either the superconducting flux transformer or a multiloop configuration. As will be shown, the results are very encouraging, and this alternative design offers significant advantages over typical ones.

## 2. Sensor Design and Fabrication Process

**Figure 1:**
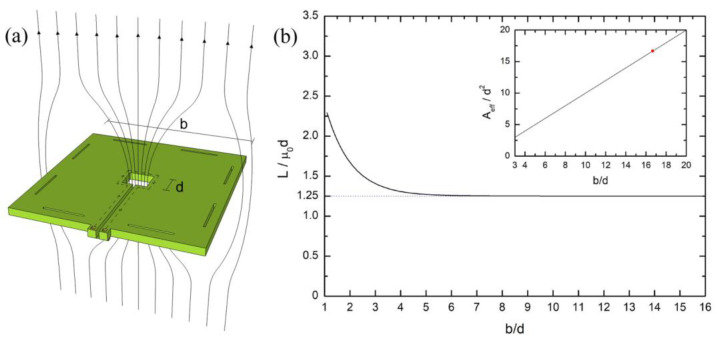
(**a**) Schematic representation of the behavior of the magnetic field flux lines in presence of a hole in a large washer-shaped coil. (**b**) Washer inductance calculations as a function of b/d ratio performed by using the software InductEx lite 2015 developed by the company Sun Magnetics (Stellenbosch, South Africa) (*b* is the washer external dimension and d is the hole dimension). The inductance value becomes approximately independent from the external dimension starting from *b* three times greater than *d*. The inset shows the estimated increase in dc SQUID effective area with respect to the geometrical area (*d*^2^) as a function of the *b*/*d* ratio. The red dot indicates our design condition: *b*/*d* = 16.7.

The dc SQUID magnetometer design simply consists of a large washer-shaped loop interrupted by two Josephson junctions. In other words, the dc SQUID ring consists of a single coil made of a superconducting square structure including a hole that has a small side length *d* with respect to the outer dimensions *b* (Figure 1a). In such a way, due to Meissner’s effect, the magnetic flux lines are focused inside the central hole (focusing effect), as reported schematically in Figure 1a. This implies that the screening currents essentially flow along the hole edges making the dc SQUID inductance insensitive from the outer dimension. Indeed, if the coil width *w* of the washer is greater than the hole size *d*, the inductance can be calculated only by considering the hole dimension. In particular, as shown in Figure 1b, the washer inductances can be calculated as *L* ≅ 1.25 *µ*_0_*d* (µ_0_ is the vacuum magnetic permeability) already starting from *b* > 3*d*, i.e., as soon as the coil width exceeds the hole dimension [28,29,30,31]. Theoretically, one can realize a dc SQUID washer wide enough to obtain a suitable effective area to increase the magnetic field sensitivity. Unfortunately, to insert the Josephson junctions, a slit that completely cuts one side of the washer needs to be inserted with the consequent dispersion of the magnetic field threading the superconducting loop. So, the screening current path becomes like the one shown in Figure 1a, giving rise to an additional inductance, which increases with the slit length *ℓ*, and reducing the possibility to extend the outer washer dimension at will. Hence, the total inductance can be written as follows [31]:

(1) $$L=1.25\mu_{0}d+L_{sl}lL_{sl}=0.3\;[\frac{pH}{\mu m}]$$

As mentioned above, the flux focusing effect makes the effective area of the dc SQUID greater than the geometrical one. The effective area can be evaluated by considering the behavior of the screening currents in the superconducting washer without the slit in the presence of an applied magnetic field superposed with the case of the magnetic flux trapped in the hole in the absence of the applied magnetic field. The result for a square washer, again under the hypothesis that the washer width is larger than the hole dimension, can be resumed as follows [28]:

$$A_{eff}=kbd$$

**Figure 2:**
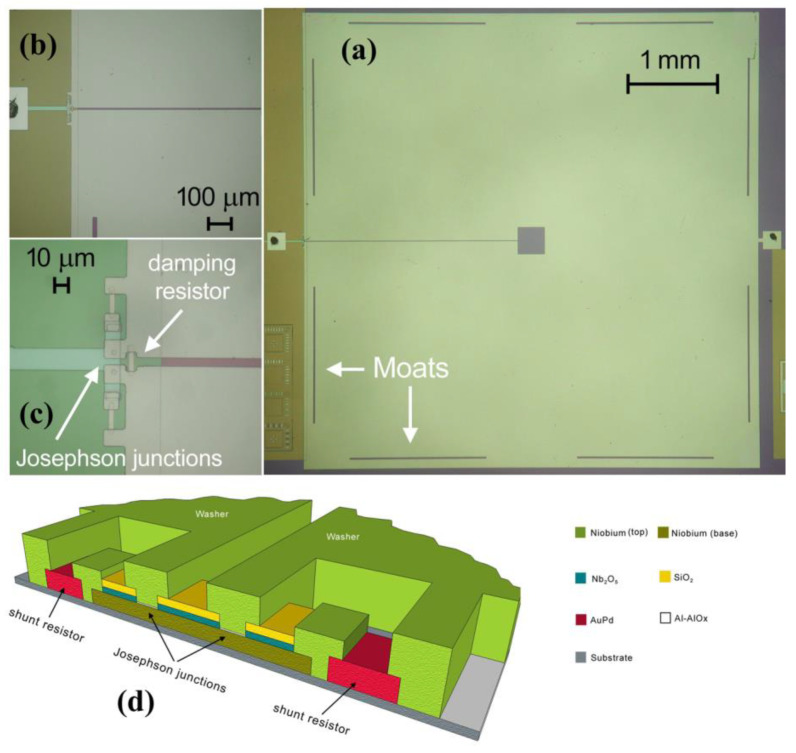
Pictures of dc SQUID magnetometer. (**a**) The large washer and the moats near the edge to trap magnetic vortices are visible. (**b**,**c**) Two details showing the Josephson junctions, the shunt resistors, and the damping resistor. (**d**) Cross section scheme of the junctions, shunt resistor, and the washer.

Here, *k* is a numerical constant close to the unity. So, the effective area increases with respect to the geometrical one by a factor equal to the *b*/*d* ratio, as reported in the inset of the Figure 2b. In our case, the *b*/*d* ratio is 16.7; therefore, the effective area of the dc SQUID washer is 16.7 times the geometrical area of the washer hole (d^2^). Of course, the effective washer area must also include the effective area of the slit *A_sl_*. A similar design based on a large washer with 6 × 6 mm^2^ and 8 × 8 mm^2^ dimensions was also used to realize YB_2_C_3_O_7_ rf SQUIDs based on step-edge junctions [32].

Considering the values reported in Table 1, we obtain an inductance value equal to 1.18 nH and an effective area greater than 2 mm^2^, which corresponds to a magnetic field–flux conversion factor (B_Φ_) less than 1 nT/Φ_0_, i.e., a magnetic field less than 1 nT is needed to couple a magnetic flux quantum Φ_0_ into the dc SQUID loop. As mentioned previously, the large slit length leads to an inevitable increase in the total inductance, which is approximately five to ten times greater than the typical values used in dc SQUID magnetometers based on superconducting flux transformers [21,22]. Consequently, the inductance parameter β_L_ = 2LI_0_/Φ_0_ increases (I_0_ is the Josephson critical current of the single junction), causing a degradation of the characteristics of the dc SQUID such as the voltage–magnetic flux (V-Φ) characteristic and the transfer factor V_Φ_, which decreases with the β_L_ value. In our case, considering a critical current of 9.1 μA, a β_L_ = 10 can be estimated. However, based on the simulations reported in [33,34], if a sufficiently small damping resistor is inserted in parallel with the inductance of the dc SQUID, the characteristics of the device are not degraded even for a large β_L_ value. In particular, the simulations show that if the ratio between the shunt resistor (R_s_) and the damping resistor (R_d_) γ = R_s_/R_d_ ≅ 1, the V-Φ characteristic and V_Φ_ are almost independent of the β_L_ value [33]. As regards the noise characteristics, in the case of a large β_L_ value, the damping resistor seems to improve them considerably. In particular, the spectral density of the magnetic flux noise of a dc SQUID with a β_L_ = 10 is only about four times larger than β_L_ = 1, while in the absence of damping (γ = 0), the magnetic flux noise is over twenty times greater [34].

To prevent low frequency noise due to the motion of the magnetic vortices inside the body of the washer, eight moats were inserted, with two on each side. The moats act as a trap for the magnetic vortices and prevent their motion; in this way, they reduce or eliminate the low frequency magnetic flux noise [35,36].

The choice of the dimensional parameters of the dc SQUID magnetometer was the result of a compromise between having an adequate B_Φ_ field sensitivity (less than 1 nT/Φ_0_) and not excessively increasing the inductance of the sensor, which leads to a degradation of its performance.

Compared to traditional designs, this is much simpler from a fabrication point of view, bringing a series of advantages. First, the fabrication yield is certainly higher: in the case of a superconducting flux transformer, in fact, an accurate electrical insulation must be provided between the input coil and the washer. Even if during the first test, the insulation appears to be good, it is not excluded that during the various thermal cycles of the device, the insulating layer may suffer small lesions, which compromise the insulation; therefore, the effective area of the sensor is drastically reduced. Even the superconductor film of the pick-up coil, following numerous thermal cycles, may have fractures, with the inevitable consequence of losing its functionality. Finally, as it is known, the presence of the input coil could induce resonances in the voltage–magnetic flux characteristics, degrading the performance of the device in terms of both noise and stability. The disadvantages of the design proposed here, despite the damping effect on the dc SQUID inductance, are essentially the low value of the current modulation depth (ΔI_S_), i.e., the difference between the critical current for the applied flux Φ = Φ_0_ and Φ = Φ_0_/2. Such a current modulation depth is related to the output voltage of the dc SQUID (ΔV): a decrease in ΔI_S_ leads to a decrease in ΔV. It is therefore necessary to increase the shunt resistance of the junctions to increase the amplitude of the voltage ΔV and the transfer factor V_Φ_. This implies that we need to work in a regime that is not too far from the hysteresis of the device which occurs when the Stewart–McCumber parameter β_C_ = 2πI_0_CR^2^_s_/Φ_0_ is greater than one (C is the capacitance of the single Josephson junction). However, thanks to very reliable and reproducible thin film deposition techniques, it is not difficult to fabricate integrated resistors whose resistance has a value with an accuracy of a few percent. Alternatively, we could think to increasing the Josephson critical current to have the same effect on the ΔV and V_Φ_, but in this case, we will further increase the β_L_, which is already large due to the design strategy used.

The dc SQUID magnetometer has been made of Niobium, a refractory material, by exploiting well established fabrication technology [37,38]. Such a fabrication process allows us to obtain Josephson junctions showing a quality factor (V_M_) greater than 80 mV at T = 4.2 K. A picture of a dc SQUID magnetometer is reported in Figure 2.

## 3. Results and Discussion

The magnetometer characterization has been carried out in a liquid helium bath at T = 4.2 K by using a cryogenic insert provided with a sample holder that has a double magnetic shield arranged as concentric cylinders made of lead and µ-metal. The low noise readout electronics are housed in a copper box kept at room temperature. The electrical connections between the two stages are suitably radio frequency filtered. The measurement of the magnetic flux noise is based on a direct coupling readout scheme including a negative feedback circuit, known as a flux locked loop (FLL), to linearize the dc SQUID output and increase the linear dynamic range [39,40]. The voltage and current noise of the readout amplifier is S_V_^1/2^ = 0.7 nV/Hz^1/2^ and S_I_^1/2^ = 2 pA/Hz^1/2^, respectively. In order to apply the excitation magnetic field to the dc SQUID magnetometer, a Helmholtz coil with a diameter of 4 cm has been employed to ensure a uniform magnetic field across the area of the sensor. A triangular current signal at a frequency of 10 Hz was sent into the Helmholtz coil to generate the magnetic field.

**Figure 3:**
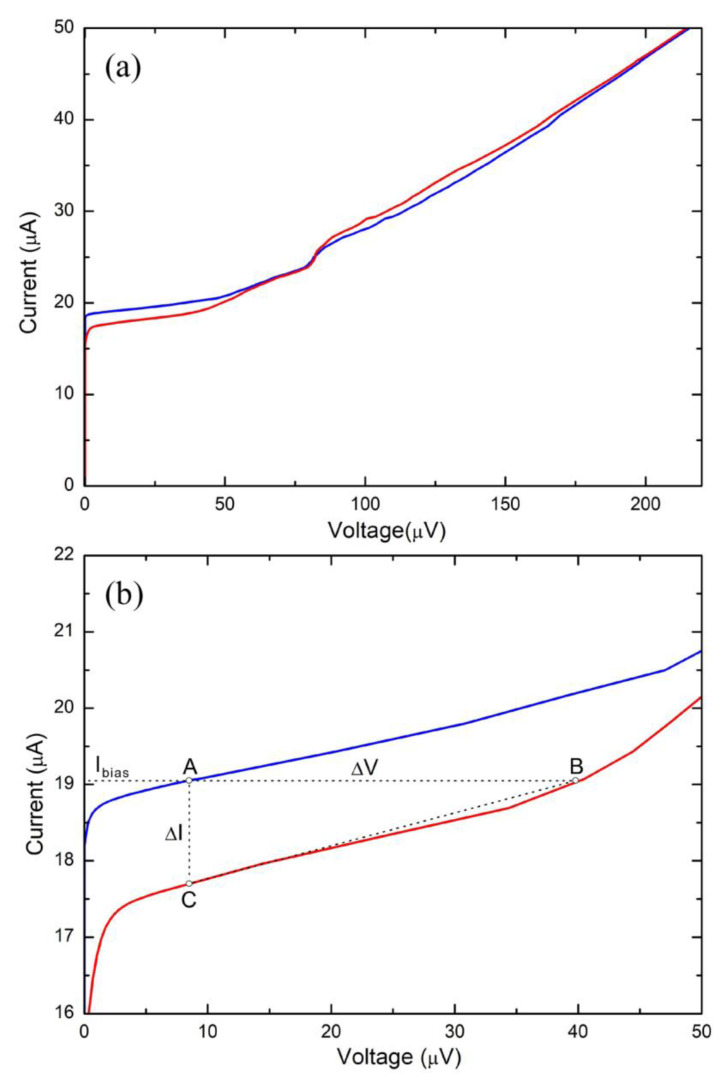
(**a**) Current–voltage (I-V) characteristic of the dc SQUID magnetometer measured at the temperature of liquid helium (T = 4.2 K) for two different values of the external magnetic flux: Φ = Φ_0_ (blue line) and Φ = Φ_0_/2 (red line). (**b**) Detail of the current–voltage characteristic from which it is possible to evaluate the dynamical resistance, the critical current modulation depth, and the dc SQUID voltage output.

Figure 3 shows the current–voltage (I-V) characteristic of the dc SQUID magnetometer measured at the temperature of liquid helium (T = 4.2 K) for two different values of the external magnetic flux: Φ = Φ_0_ and Φ = Φ_0_/2. To distinguish the two curves, we used two different color lines: the blue line for the I-V measured at Φ = 0 and the red line for the I-V measured at Φ = Φ_0_/2. As expected, it is evident the rounding of the characteristics closes to the zero-voltage state due to thermal noise [38,39]. The critical current is equal to I_S_ = 2I_0_ = 18.2 μA and the resistance R_NN_ = R_s_/2 measured in the linear region of the I-V characteristic (above 100 μV) is 4.7 Ω, from which Rs = 9.4 Ω, which is in perfect agreement with the designed value (9.5 Ω). The modulation depth of the critical current ΔI_S_ is equal to 2.4 μA; therefore, the ratio ΔI_S_/2I_0_ ≅ 0.13 which, compared with the theoretical curves, corresponds to a β_L_ value of approximately 5 [41]. It is worth noting that the β_L_ value calculated with the parameters reported in Table 1 is approximately double compared to that obtained previously through the experimental ratio ΔI_S_/2I_0_. A smaller β_L_ value leads to an increase in the current modulation depth, voltage swing, and transfer factor, as well as a better performance in terms of magnetic noise. This could be explained in the framework of dc SQUID simulations with a damping resistance [33,34]. Considering a capacity of the single junction of about 1.0 pF, a Stewart–McCumber parameter β_C_ of 2.5 is obtained, while the noise parameter is Γ = 2πk_B_T/I_0_Φ_0_ = 0.020, where k_B_ = 1.38 × 10^−23^ J/k is the Boltzmann constant. The capacitance of the junction was evaluated via Fiske steps measurements [42] on junctions fabricated in the same batch as the SQUID magnetometers. Thanks to the presence of thermal noise, there is no hysteresis in the characteristic of the device, as can be seen from Figure 3, despite the high β_C_ value [43,44]. At the same time, there is a high dynamic resistance R_dyn_ for voltages close to zero, which leads to the desired effect of increasing the voltage swing ΔV and the transfer factor V_Φ_. In fact, from Figure 3b, a R_dyn_ ≅ 24 Ω can be measured by taking the ratio between the ΔV and ΔI:

(2) $$R_{dyn}={\left(\frac{\partial I}{\partial V}\right)}_{Ibias}^{-1}≅\;{\left(\frac{∆I}{∆V}\right)}^{-1}\;∆V≅R_{dyn}∆I\;$$

ΔI = I_A_ − I_C_ = 1.3 μA is the difference between the current values at Φ = 0 and Φ = Φ_0_/2 (points A e C in the Figure 3b), evaluated at the bias current point I_bias_ = 19 μA, just above the dc SQUID critical current, while ΔV = 31 μV is given by V_B_ − V_A_, where A and B (indicated in Figure 3b) are the points in which the straight line parallel to the voltage axis (I_bias_ = cost) intercepts the two curves for Φ = 0 and Φ = Φ_0/2_.

The approximation in (2) is reasonable because the straight line passing through B and C is approximately equal to the straight-line tangent to the I-V curve at the bias point. The bias point is chosen to optimize the amplitude and the shape of the V-Φ characteristics; typically, the optimal value is I_bias_ = 2.1I_0_. Note that the I-V curves differ from those corresponding to the RSJ (Resistively Shunted Junction) model and there is an undamped resonance corresponding to a voltage of about 70 μV. As expected from the theoretical simulations [44,45], this is due to a high β_C_ value, despite the presence of the damping resistor. However, as can be seen from the I-V characteristics, the resonance is quite far from the working point of the sensor and therefore does not create any problems. Due to the presence of the resonance, the two I-V curves for Φ equal to 0 and Φ_0_/2 intersect, and the relative bias current value (about 26 μA) corresponds to the first zeroing of the ΔV in the V-Φ characteristics. Overcoming this bias current value, the ΔV grows again and for sufficiently high bias currents (greater than 45 μA), the V-Φ characteristic disappears definitively.

**Figure 4:**
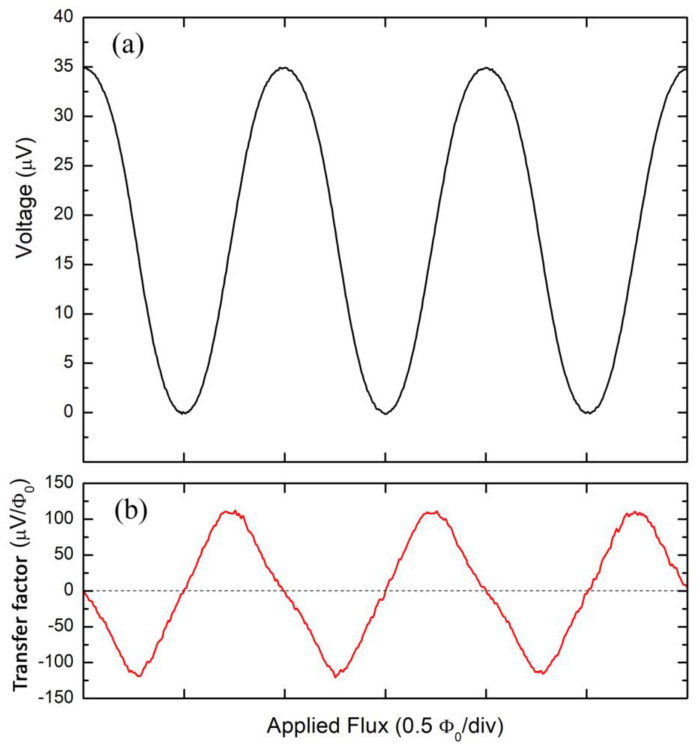
(**a**) Voltage–magnetic flux characteristics (V–Φ) measured at T = 4.2 K of a dc SQUID magnetometer. One Φ_0_ corresponds to 0.82 nT, so the effective flux capture area is 2.5 mm^2^. (**b**) Transfer factor V_Φ_ as a function of the external magnetic flux obtained by taking the derivative of the V-Φ characteristics. The maximum V_Φ_ value is 110 μV/Φ_0_ obtained for a Φ = Φ_0_/4.

In Figure 4a, the voltage and transfer factor as a function of the external magnetic flux are reported. They have been measured at T = 4.2 K for I_bias_ = 19 μA. The voltage swing is ΔV = 31 μV, which is in accordance with the estimated value by the I-V characteristics. The magnetic field–flux conversion factor B_Φ_ measured using a uniform field generated by the Helmholtz coil is 0.82 nT/Φ_0_ and corresponds to an effective area of 2.5 mm^2^. Considering that the effective area of the washer is 1.5 mm^2^, to which the effective area of the slit (about 0.8 mm^2^) must be added, the agreement is quite good.

Figure 4b shows the transfer factor V_Φ_ as a function of the external magnetic flux obtained by taking the derivative of the V-Φ characteristics. The maximum value of the transfer factor V_Φ_ estimated in Figure 4b is 110 μV/Φ_0_, corresponding to an external magnetic flux value equal to approximately Φ_0_/4. In the case of the electronic readout based on a direct coupled scheme, this implies a magnetic flux noise electronic contribution equal to S_Φ,el_^1/2^ = S_V,el_^1/2^ /V_Φ_. For a low noise amplifier with a S_V_ = 0.7 nV/Hz^1/2^ as in our case, we have a S_Φ,el_ ≅ 7 μΦ_0_/Hz^1/2^ that must be taken into account for the estimation of the intrinsic noise of the quantum sensor. In any case, this additional noise term can be significantly reduced by using an additional positive feedback circuit (APF) which distorts the V-Φ characteristics by increasing the slope, i.e., the transfer factor in one of the sides of the characteristic [39,40]. It is important to note that the characteristics are free of resonances or other structures and are very smooth, ensuring the excellent stability of the working point.

**Figure 5:**
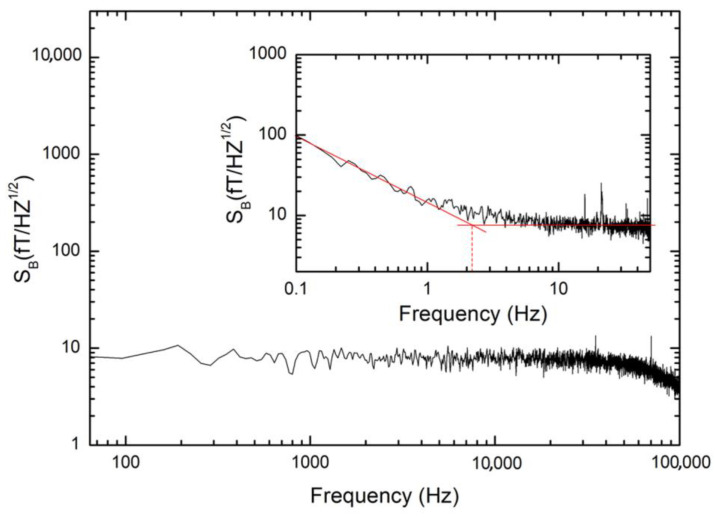
Field noise spectral density of the investigated magnetic sensor measured at 4.2 K using a direct-coupled scheme with a low noise voltage amplifier. The white magnetic noise is 8 fT/Hz^1/2^ with a knee of low frequency noise of 2 Hz, which is essentially due to amplifier noise (inset). The intersection of the two red lines gives us the knee value of the low frequency noise.

In Figure 5, the intrinsic spectral density of the magnetic field noise S_B_^1/2^ of the quantum magnetometer measured at T = 4.2 K is reported by using a flux locked loop scheme (FLL) [39,40]. As shown in the figure, the white noise is 8 fT/Hz^1/2^, down to 2 Hz, below which the low frequency noise is mainly due to the amplifier noise. This spectrum was obtained by multiplying the values of the magnetic flux spectrum with the B_Φ_ value (S_B_^1/2^ = S_Φ_^1/2^ B_Φ_); so, considering a B_Φ_ value = 0.82 nT/Φ_0_, the spectral density of the magnetic flux noise is 9.8 μΦ_0_/Hz^1/2^, which is comparable with the theoretical estimates. In fact, considering the theoretical simulations [41] for β_C_ values different from zero, β_L_ = 1 and I_bias_ = 2.1 I_0_, we can obtain the following:

(3) $$S_{\Phi }^{1/2}≅9\;\sqrt{\frac{k_{B\;}T}{I_{0}^{2}\;R}}\;\Phi_{o}$$

Substituting the numerical values, we obtain S_Φ_^1/2^ ≅ 2.5 μΦ_0_/Hz^1/2^. In our case, β_L_ = 5 and Γβ_L_ = 0.1, we have to multiply by a factor of four, as predicted by the numerical simulations [41]. We therefore obtain S_Φ_^1/2^ ≅ 10 μΦ_0_/Hz^1/2^, which is in good agreement with the measured value. On the other hand, Enpuku’s simulations [34] predict that a value of β_L_ = 10 leads to an increase in magnetic flux noise of only four times the value relative to β_L_ = 1.

It is worth noting that a magnetic sensor with a noise of 8 fT/Hz^1/2^ can be employed for all applications, including those of magnetoencephalography, since it works stably in a FLL scheme and has a low level of 1/f noise. Furthermore, if we calculate the energy resolution per bandwidth unit by using the formulas ε = S_Φ_/2L, we obtain a value of approximately 70 *h* (where *h* is the Plank constant), which is more or less the same obtained with a classic dc SQUID magnetometer with a flux noise of 2–3 μΦ_0_/Hz^1/2^ and an inductance of 100 pH. Since ε is the parameter for comparing dc SQUIDs with different designs, we can state that, in terms of performance, the proposed quantum magnetometer is equal to classical magnetometers that use additional complicated superconducting circuits, but at the same time it offers the numerous advantages that we have previously explained.

To test the robustness of the sensors with respect to thermal stress, many thermal cycles were performed from room temperature directly to helium liquid temperature (4.2 K), and the characteristics of the sensors within the instrumental experimental uncertainties did not change, nor did we observe sensors that no longer worked. Furthermore, to verify reproducibility, several devices belonging to different fabrication batches were measured, showing very similar performances to each other.

## 4. Conclusions

In conclusion, a dc SQUID magnetometer based on an alternative design to the typical ones and usable for high-sensitivity applications has been designed, fabricated, and characterized at liquid helium temperature. The basic idea was to use the flux focusing effect in a washer-shaped dc SQUID with a size that would guarantee enough of an effective area (about 2.5 mm^2^) to allow high-sensitivity magnetometry applications. A suitable damping of the SQUID inductance and an appropriate choice of fabrication parameters prevented us from degrading the performance of the sensor in terms of magnetic flux noise and the amplitude of the voltage–magnetic flux characteristics. The experimental results show us that it is possible to obtain a bare dc SQUID with a magnetic field sensitivity less than 8 fT/Hz with a low frequency noise knee less than 2 Hz. The simplicity of the design allows us to obtain a high-performance quantum sensor that is very robust, stable, and reliable. Since there are no additional circuits, such as the superconducting flux transformer, fabrication is less critical, and results in a better yield. Furthermore, there are no resonances that degrade the performance of the device and make it less stable and noisy. We would like to stress that the performance of the SQUID magnetometer proposed here is similar to the one based on the superconducting flux transformer and has approximately the same dimensions [46]. These results are original and significant and certainly of interest to the large community carrying out research on quantum sensors and their applications.

## Figures and Tables

**Table 1:** Design parameters of the superconducting quantum magnetometer.

Design Parameters	
Josephson Junctions	
Critical current	I_0_ = 9.1 μA
Area	A = 16 μm^2^
McCumber parameter	β_c_ = 2.5
Capacitance	C = 1.0 pF
Shunt resistance	R_s_ = 9.4 Ω
Noise parameter	Γ = 0.02
dc SQUID Washer	
Outer dimension	b = 5 mm
Hole dimension	d = 0.3 mm
Slit length	b_T_ = 2.35 mm
Slit width	w = 8 μm
Hole inductance	L_h_ = 472 pH
Slit Inductance	L_T_ = 705 pH
Moats	
Number	Nr = 8
Dimensions	A = 20 × 1500 μm^2^

## Data Availability

Data is contained within the article.
